# Draft Genome Sequence of *Mycobacterium mageritense* DSM 44476^T^

**DOI:** 10.1128/genomeA.00354-14

**Published:** 2014-05-01

**Authors:** Olivier Croce, Catherine Robert, Didier Raoult, Michel Drancourt

**Affiliations:** Aix Marseille Université, URMITE, Marseille, France

## Abstract

We report the draft genome sequence of *Mycobacterium mageritense* strain DSM 44476^T^ (CIP 104973), a nontuberculosis species responsible for various infections. The genome described here is composed of 7,966,608 bp, with a G+C content of 66.95%, and contains 7,675 protein-coding genes and 120 predicted RNA genes.

## GENOME ANNOUNCEMENT

*Mycobacterium mageritense* has been initially reported as a new species of the *Mycobacterium fortuitum* complex (1) based on the description of five isolates from the respiratory tract of five unrelated patients in Spain (2). The analysis of the partial *rpoB* gene sequence, however, did not confirm such a taxonomic assignment (3). Refined analysis incorporating the sequence of the 16S rRNA gene and four housekeeping genes indicated that *M. mageritense* stands by itself outside any known mycobacterial complex (4). Further isolates were made from the respiratory tract (5–7), blood obtained from patients withf catheter-related infections (5, 8), cerebrospinal fluid in patients with intrathecal catheters (9), sinus drainage, and surgical wound infections (5).Two isolates were obtained from cutaneous lesions in women who received a pedicure and whirlpool footbath from which *M. mageritense* was also recovered (10). In addition, *M. mageritense* has been recovered from cutaneous lesions of a tsunami survivor (11). Environmental isolates have been made from soil in Japan (12).

We aimed to contribute to the determination of the taxonomic relationships of *M. mageritense* by sequencing the whole genome of the *M. mageritense* DSM 44476^T^ strain.

Genomic DNA was isolated from the *M. mageritense* DSM 44476^T^ strain grown on Mycobacteria Growth Indicator Tube (MGIT) Middlebrook broth at 37° C. Genomic DNA of *M. mageritense* was sequenced on the MiSeq Technology (Illumina, Inc., San Diego, CA) with the two applications: paired end and mate pair, in a 2- × 250-bp run for each bar-coded library. On each flowcell, the index representation for *M. mageritense* was determined to 5.09 and 7.11%, respectively. The global 1,572,948 reads were filtered according to the read qualities.

Illumina reads were trimmed using Trimmomatic (13), then assembled with Spades software (14, 15). Contigs obtained were combined by using SSPACE (16), Opera software v 1.2 (17), and GapFiller v 1.10 (18) to reduce the set. Some manual refinements using CLC Genomics v 6 software (CLC bio, Aarhus, Denmark) and homemade tools improved the genome sequencing. The final draft genome of *M. mageritense* consists of six contigs, containing 7,966,608 bp and a G+C content of 66.95%.

Noncoding genes and miscellaneous features were predicted using RNAmmer (19), ARAGORN (20), Rfam (21), and PFAM (22). Open reading frames (ORFs) were predicted using Prodigal (23), and functional annotation was achieved using BLASTP against the GenBank database (24) and the Clusters of Orthologous Groups (COGs) database (25, 26). The genome was shown to encode at least 120 predicted RNAs, including 4 rRNAs, 95 tRNAs, 1 tmRNA, and 20 miscellaneous RNAs. A total of 7.675 genes were identified, representing a coding capacity of 7,385,502 bp (coding percentage, 92.7%). Among these genes, 7,615 (99.2%) genes matched a least one sequence in the COGs database with BLASTP default parameters, 972 (12.66%) encode putative proteins, and 1,431 (18.64%) were assigned as hypothetical proteins.

### Nucleotide sequence accession numbers.

The *Mycobacterium mageritense* strain DSM 44476^T^ genome sequence has been deposited at EMBL under the accession numbers CCBF010000001 through CCBF010000006.
